# A case series to test the acceptability, feasibility and preliminary efficacy of AVATAR therapy in anorexia nervosa

**DOI:** 10.1186/s40337-023-00900-1

**Published:** 2023-10-13

**Authors:** Alistair Thompson, Chiara Calissano, Janet Treasure, Hannah Ball, Alice Montague, Thomas Ward, Valentina Cardi

**Affiliations:** 1 Department of General Psychology, University of Padova, Padova, Italy; 2 Department of Psychological Medicine, Institute of Psychiatry, Psychology and Neuroscience, King’s College London, London, SE5 8AF UK; 3 Division of Psychology and Mental Health, School of Health Sciences, Faculty of Biology, Medicine and Health, The University of Manchester, Manchester, M13 9NT UK; 4 Manchester Mental Health NHS Foundation Trust, Manchester, M25 3BL UK; 5 North East London Foundation NHS Trust, London, E17 3HP UK; 6 Department of Psychology, Department of Clinical, Educational, and Health Psychology, University College London, London, WC1E 7HB UK; 7 Department of Psychology, Institute of Psychiatry, Psychology and Neuroscience, King’s College London, London, SE5 8AF UK; 8 South London and Maudsley NHS Foundation Trust, London, UK

**Keywords:** Anorexia nervosa, AVATAR, Distress, Eating disorders, Feasibility, Self-compassion, Single-case experimental design, Thematic analysis, Voice

## Abstract

**Background:**

Patients with anorexia nervosa tend to experience an inner “eating disorder” voice. They struggle to recognise and assert their own identity over the illness’s identity and relate to it from a powerless and subordinate position. AVATAR therapy was developed to help patients with psychosis to gain greater power and control over distressing voices. The goal of this study was to test the feasibility, acceptability, safety and preliminary efficacy of an adaptation of AVATAR therapy for anorexia nervosa.

**Methods:**

Twelve adult patients with anorexia nervosa were recruited. Ten completed an assessment session and between five to seven therapy sessions. The assessment session consisted in the creation of an avatar to represent the “eating disorder”. This was accomplished by manipulating auditory and visual characteristics through a specialist computer software. During the therapy sessions, patients interacted with the avatar to assert their own desires and will. Patients completed baseline, end of intervention and follow-up (4-week) online questionnaires. A non-concurrent multiple baselines single case experimental design (SCED) was used (A_1_BA_2_). Feasibility, acceptability, safety and preliminary efficacy of the intervention were assessed.

**Results:**

The therapy met pre-specified criteria relating to (1) Feasibility: sample recruited within three months; retention rate at the end of the treatment phase = 81.9%; therapy completion rate = 90.1%. (2) Safety: no serious adverse events associated with the intervention. (3) Acceptability: mean ratings = 7.5 (*SD* = 2.61) out of ten, on a 0–10 scale of acceptability (10 = complete satisfaction). With regards to efficacy, participants reported significantly lower levels of distress associated with the eating disorder voice and higher levels of self-compassion at the end of treatment. No other significant changes were observed in frequency of the eating disorder voice, voice’s characteristics, such as omnipotence and malevolence, eating disorder symptoms and symptoms of anxiety, depression and stress. Patients’ feedback indicated that the therapy had helped with their ability to stand up to the illness, make positive changes around eating, and increase their motivation to recover and self-compassion.

**Conclusion:**

AVATAR therapy for anorexia nervosa is feasible, acceptable and safe for patients. Larger studies are needed to test clinical efficacy.

***Trial registration*:**

The study was pre-registered on the clinicaltrials.gov registry (https://clinicaltrials.gov/ct2/show/NCT04778423).

**Supplementary Information:**

The online version contains supplementary material available at 10.1186/s40337-023-00900-1.

## Abstract (plain language summary)

People with anorexia nervosa often struggle with connecting to their identity beyond the illness. They tend to relate to the eating disorder from a powerless and submissive position. The aim of this study was to test AVATAR therapy for anorexia nervosa. The goal of the intervention was to help patients to assert themselves, their identity, will, power and desires, when confronted with the “voice” of the illness. Twelve patients with anorexia nervosa were recruited and 10 completed one assessment session and five to seven therapy sessions. During the assessment session, patients were guided by the therapist to create a representation of their illness (“the avatar”) by manipulating visual and auditory characteristics through a computer software. During treatment, they were supported by the therapist to assert themselves over the illness, responding to the abusive and critical comments of the eating disorder delivered by “the avatar”. Patients’ qualitative feedback indicated that the therapy was meaningful to them, and that it helped to recognise their own voice as distinct from the illness. The distress perceived in relation to the eating disorder voice decreased over time, and feelings of self-compassion increased at the end of therapy. Findings point towards the acceptability and usefulness of AVATAR therapy for anorexia nervosa. Larger studies are needed expand these results.

**Supplementary Information:**

The online version contains supplementary material available at 10.1186/s40337-023-00900-1.

## Background

Anorexia nervosa is marked by dangerous eating and weight control behaviours. It is one of the most challenging mental disorders to treat, with 20% of patients developing a chronic illness and mortality rates ranging from 6 to 15% [1]. Psychological treatment is recommended by international guidelines [2]. However, there is limited evidence for the superiority of one specific intervention over another, with the exception of family-based therapy for younger patients, which seems to outperform active control conditions [3]. Even when valid treatment options are offered, treatment acceptability remains a problem, as evidenced by drop-out rates ranging from 20 to 70% in outpatient settings [4] and qualitative feedback from patients signalling ongoing psychological distress following weight restoration (perceived as the main treatment focus; 5). This evidence indicates the urgent need for novel interventions which are acceptable across diverse service users and designed to target a broader range of relevant psychological factors in addition to abnormal eating, weight, and shape-control behaviours.

One of the most important barriers to recovery, identified by patients, is the difficulty in developing an identity beyond the illness [5, 6]. In the acute phase of anorexia nervosa, the illness’s identity often co-exists with the individual’s weakened sense of self and it is referred to as the “eating disorder voice” [7, 8]. Patients commonly describe a form of relationship between these two different parts of the self (the eating disorder and themselves) and highlight qualitative changes over time. Early on, the sense of self provided by the illness is often considered helpful in achieving personal goals (e.g., sense of control and desired change in weight and appearance). This positive relationship between the self and the illness defines the egosyntonic and valued nature of the eating disorder in this phase. However, as the illness progresses the relationship can become more distressing, as personal qualities are undermined and the true self is silenced [9, 10]. Individuals commonly report a sense of powerlessness and loss of identity as anorexia nervosa becomes an internal voice which dominates the self [8, 10]. This is why one of the main goals of treatment is to help patients externalising the illness (i.e., perceiving the disorder as something separate from the self). By recognising the illness as a separate entity, patients are encouraged to “feel” the consequences of the disorder, to fight it and challenge its commands, while recognising, reinforcing and holding on their own values and desires, as individuals [11].

While the relationship with the eating disorder voice typically changes over time and across stages of the illness, when patients with eating disorders experience the voice as hostile and malevolent, they tend to relate to it from a position of perceived subordination and powerlessness, similarly to how people with psychosis relate to their own voices [12]. Audio Visual Assisted Therapy Aid for Refractory auditory hallucinations (“AVATARtherapy”) is a novel approach specifically developed in psychosis to target the relationship between the voice-hearer and the voice. In AVATAR therapy, the voice-hearer is first supported to create a computerised representation of the distressing voice (“the avatar”), matched with respect to auditory and visual characteristics. The therapist then facilitates a series of real-time dialogues between the individual and the main voice that they hear, using a bespoke voice transformation software [13]. The dialogue is aimed at asserting power and control over the voice-hearing experiences. Following an initial pilot trial [13], a fully powered randomised trial of AVATAR therapy has demonstrated large treatment effects (*d* = 0.8), against an active control, in reducing the severity of auditory hallucinations in participants identified as treatment-resistant [14]. A recent systematic review [15] has presented encouraging evidence for the effectiveness of AVATAR therapy by researchers working independently from the creators of the original protocol.

In recent years, our research group has been collaborating with experts in AVATAR therapy for psychosis, alongside people with lived experience of eating disorders, to adapt the approach to patients with anorexia nervosa. The overall goal was to develop a therapy through which individuals could empower themselves over the illness and re-gain a sense of self or recovery identity. The first step of this preliminary work was to test the feasibility and acceptability of individuals with anorexia nervosa interacting with personalised digital avatars representing their illness. Twenty-one patients were involved, all of whom reported that the avatar was an acceptable representation of their illness and that they would be willing to have a dialogue with it [16]. Having established proof of concept, the next stage was to involve individuals with lived experience in the adaptation of the AVATAR therapy protocol to anorexia nervosa. Three recovered women and one woman who was currently ill provided written feedback on a draft version of the therapy manual. Eight currently ill patients and seven carers also took part in two separate online focus groups to answer questions related to the “eating disorder voice” and the potential of AVATAR therapy to stand up to the voice. AVATAR therapy was considered a “logical” step to challenge the illness, and a helpful way to differentiate the illness from the healthy self. Patients also highlighted feelings of fear and uncertainty as to how a “real” dialogue with the avatar might sound.

The present study was the first to explore the delivery of AVATAR therapy in anorexia nervosa and evaluated the feasibility, acceptability and safety of the approach against pre-defined and pre-registered criteria. A preliminary analysis of the effect of the therapy on illness-related distress and eating disorder psychopathology was also conducted.

## Methods

### Design

The study was pre-registered on the clinicaltrials.gov registry (https://clinicaltrials.gov/ct2/show/NCT04778423). A non-concurrent multiple baselines single case experimental design (SCED) was used (A_1_BA_2_). The choice and characteristics of this design are described in Additional file 1: Material 1. During the baseline phase (A_1_) participants were randomly allocated to a 2-, 3- or 4-week baseline. In the intervention phase (B), they received one assessment session and up to seven sessions of AVATAR therapy. Following the intervention, patients were invited to complete a 4-week follow-up (A_2_). The study was approved by the London—Fulham Research Ethics Committee (21/LO/0384) and the Health Research Authority (HRA).

### Participants

The SCED standards require to involve a minimum of three participants per baseline phase [17]. In outpatient treatment settings, the dropout rate from patients with anorexia nervosa is estimated to be between 20 and 40% [18]. The aim was therefore to recruit a minimum of 12 participants to ensure that there would be at least three participants in each baseline phase, after accounting for drop-out. Patients were recruited from a database of people who had participated in previous studies led by the research team, and who had provided consent to be re-contacted. Inclusion criteria were: (1) over 16 years old, (2) diagnosis of anorexia nervosa based on the criteria of the Diagnostic and Statistical Manual of Mental Disorders, 5^th^ edition (DSM–5; 19), (3) knowledge of English and (4) currently receiving treatment for the eating disorder and/or undertaking regular check-ins with a health professional. The reason why the latter criterion was introduced is because AVATAR therapy was delivered online and therefore no face-to-face monitoring of medical risk (e.g., weight deterioration) would have been possible. Among the participants recruited, only one reported engaging in a concurrent intensive intervention which was a day treatment programme. All the others were receiving regular check-ins with a health professional or the general practitioner. Participants with a self-reported diagnosis of psychosis, alcohol misuse disorder or substance misuse disorder were excluded.

### Measures

#### Feasibility, safety and acceptability

As described in the pre-registered protocol, feasibility was established through (1) 80% of sample (*n* = 12) recruited within 18 months, (2) 80% rate of retention within the study protocol (completion of end of intervention measures at 6–8 weeks), and (3) 80% retention rate within the treatment (completion rate, defined as completion of at least five of the six active dialogue sessions; Table 2). Safety was monitored by the therapists through recording of any adverse experiences related or unrelated to participation in the study (e.g., death, suicide attempts, hospital admissions and complaints about the therapy).

Acceptability was assessed in two ways. The first was a single question (“How satisfied were you with AVATAR therapy as a treatment to help people with an eating disorder?”) to answer using a 10-point scale (10 = complete satisfaction; 0 = no satisfaction at all). The pre-set criterion was that at least 80% of the sample would rate their satisfaction as equal or greater than 7. Acceptability was also investigated through qualitative interviews which included questions about participants' overall experience of the approach, timings and online delivery. The interview schedule is described in Additional file 1: Material 2.

#### Efficacy

As defined in the pre-registered protocol, the impact of the intervention was assessed on the following variables: eating disorder voice (i.e., the internal critical commentary on weight, appearance, and self-worth reflecting the illness identity), eating disorder symptoms, anxiety and depression, self-criticism and self-compassion.

Eating disorder voice characteristics were measured through: (1) the auditory hallucinations subscale of the Psychotic Symptom Rating Scale (PSYRATS, 20; adapted to eating disorders, 9), to assess voice-related distress and frequency weekly, through the study’s phases. This scale had acceptable reliability in the tested sample (*α* = 0.78) (9). (2) A brief 6-item eating disorder voice survey to measure content and perceived power of the voice, completed weekly through the study’s phases. The survey included one item of the Voice Power Differential scale (VPD, 21) and 5 items developed by the research team (Additional file 1: Material 3). In the current sample, the measure demonstrated acceptable internal consistency (α = 0.72). (3) The Beliefs About Voices Questionnaire revised (BAVQ-R, 22), a 35-item self-report adapted from the psychosis research to measure omnipotence, malevolence and behavioural responses to the eating disorder voice at baseline, end of treatment and follow-up [12]. In the current sample, the measure demonstrated acceptable internal consistency (*α* = 0.74).

Eating disorder symptoms were measured at baseline, end of intervention and follow-up using the Eating Disorder Examination-Questionnaire (EDE-Q, 23). The EDE-Q is a 28-item self-report to assess eating disorder psychopathology using four subscales (restraint, weight concern, shape concern, eating concern) and a global score. The EDE-Q has acceptable validity and test–retest reliability [24] and in the current sample the internal consistency was strong (*α* = 0.95).

Symptoms of depression and anxiety were measured through the Depression Anxiety Stress Scales Short Version (DASS-21, 25) at baseline, end of intervention and follow-up. The DASS-21 had strong internal consistency (*α* = 0.92) in this study.

Self-criticism and self-compassion were measured through the Self-Criticism and Self-Compassion Scale (SCSC, 26) at baseline, end of intervention and follow-up. The scale had strong internal consistency in this study (*α* = 0.88).

### Procedure

Following recruitment and eligibility assessment, participants completed the baseline questionnaires. Then, they attended one assessment session during which they created the avatar (90–120 min) [16] and 5–7 AVATAR therapy sessions (60 min/each). Sessions were online and delivered through a video conferencing platform. They were facilitated by a therapist, who included therapists working on the AVATAR2 for psychosis trial (TW. AM, HB) or Trainee Clinical Psychologists (AT & CC). Therapists with experience working with people with eating disorders provided supervision (VC, JT). All questionnaires were completed online using the Qualtrics Survey platform. Participants were invited to take part in a qualitative interview at the end of the study. The interviews lasted between 30 to 45 min and were conducted online; six interviews were carried out by a Clinical Psychologist that was not involved in delivering the intervention (VC) and three interviews were carried out by a Trainee Clinical Psychologist (CC); the latter individuals were seen by therapists other than CC.

### Intervention

The intervention was adapted from the AVATAR-brief manual for psychosis [14, 27, 28]. The computer technology was developed by the Speech, Hearing & Phonetic Sciences Department at University College London. An assessment session preceded treatment sessions [16]. The active dialogue sessions involved a pre-dialogue section, with a review of the past week and the previous dialogue and preparation for the next dialogue (e.g., role-plays); an active dialogue section with the avatar (5–10 min); and a post-dialogue section for active reflection on the dialogue. During the active dialogue, the therapist switched between their own voice (to facilitate the session and provide support) and voicing the avatar (using a voice transformation software). The therapist used a live webcam feed of the person throughout to provide encouragement and monitor their responses to the avatar. Participants had the opportunity to say “stop” or any personally agreed phrase if they wanted to stop the dialogue. A video recording of the dialogue was provided to the participant following each session and participants were advised to view this at least once before the next session. Details of each session content are provided in Additional file 1: Material 4.

### Statistical analyses

#### Quantitative data analysis

Feasibility, safety and acceptability were reported as descriptive data. In line with the literature on SCED data analyses, effect sizes, descriptive data and visual analysis were used to measure the impact of the intervention on clinical symptoms [29, 30]. The between case standardised mean difference (BC-SMD) was calculated for the weekly outcomes using the scdhlm R package [30, 31]. BC-SMD is an analogue of Cohen’s d [30] and it is consistent with visual analysis [30, 31]. Descriptive data were summarised using the Reliable (RCI) and Clinically Significant Change Indices (CSC, 32), both of which were calculated using the Leeds Reliable Change Index Calculator [33]. RCI and CSC were calculated using clinical and non-clinical sample means and standard deviations from previous research (Additional file 1: Material 5) [9, 14, 34]. RCI and CSC could not be calculated for the Brief 6-item eating disorder voice survey as no comparison samples exist. CSC was calculated using criterion A for voice-related distress and frequency, criterion C for the EDE-Q and SCSC, and clinical cut-offs suggested by Lovibond & Lovibond [25] were used for the DASS-21 [32]. Visual analysis was used to estimate changes in central tendency, trend, and variability for the weekly outcome measures [35]. Some of the quantitative findings of this study are also reported in the doctoral thesis of one of the co-authors (AT), supervised by the co-authors VC, TW and JT [36].

#### Qualitative data analysis

The interview transcripts were analysed using reflexive thematic analysis [37, 38]. Following familiarisation with the data, initial codes were generated from three interviews. Any differences and disagreements in coding and any emerging ideas for further coding were resolved and discussed by the two researchers conducting the analysis (CC & DCB). A coding framework was developed by coding three further transcripts and then applied to all remaining interviews. Themes were created by collating the codes, which gathered all data relevant to each potential theme. Clear definitions and labels for each theme were generated. The software NVivo (Release 1.0) for Windows was used to manage the interview data. The qualitative analysis was conducted in line with the COnsolidated criteria for REporting Qualitative research (COREQ) Checklist [39]. Data were openly coded without a pre-existing coding frame, following a primarily inductive approach. However, a degree of deductive analysis was used to ensure that the coding produced themes that were meaningful to the research questions [40]. This was informed by existing literature on AVATAR therapy and experience of the illness in individuals with anorexia nervosa. Some of the qualitative findings of this study are also reported in the doctoral thesis of one of the co-authors (CC), supervised by the co-authors VC, TW and JT [41].

## Results

### Sample characteristics

**Fig. 1:**
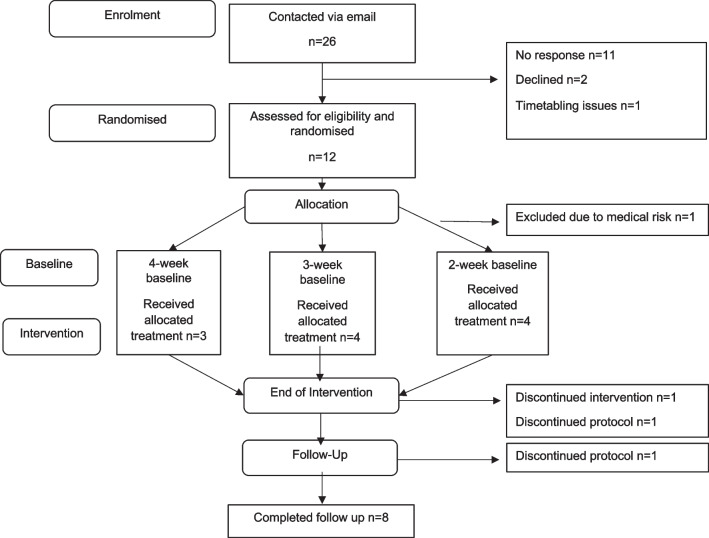
Flow of participation in the study

Twenty-six people with anorexia nervosa were contacted via email from previous studies, of whom 12 agreed to eligibility screening.. One participant never started AVATAR therapy because their Type 1 diabetes became unstable and in need of urgent medical attention and treatment. Eleven people met the eligibility criteria and were allocated to a 2- (*n* = 4), 3- (*n* = 4), or 4-week (*n* = 3) baseline. End-of-intervention assessments were completed by 10 participants and follow-up questionnaires were completed by 8 (see Fig. 1 for the flow of participation). The mean age was 27 years old (SD = 7.8, Range 18–44); 75% of the sample were of female gender (N = 9) and most participants where from a white ethnic background (91.7%). The mean duration of illness was 11.8 years (SD = 11.8, range 1–28) and the mean body mass index was 16.7 (SD = 2.3, range 14–20.28). Seven people reported a psychiatric comorbidity (depression, anxiety disorders, obsessive–compulsive disorder) but only two were taking psychiatric medication.

### Feasibility

The target sample (*n* = 12) was recruited within three months, between the 23rd July 2021 and the 3rd October 2021. (2) Retention in the study protocol at the end of treatment was 81.9%. (3) The therapy completion rate was 90.1%; one participant dropped-out from the intervention after two active dialogue sessions and provided no reason for doing so.

### Safety

No serious adverse events associated with the intervention (e.g., deaths, suicide attempts, serious violent incidents, formal complaints, crisis referrals or hospital admissions) were recorded. Further details with regards to participants’ safety are provided in Additional file 1: Material 6.

### Acceptability

Ten participants (90.1% of the sample) rated the acceptability of the therapy on a 0–10 scale (*M* = 7.5, *SD* = 2.61; range 0–9) and nine provided scores equal or greater than 7 (81.8%). One participant rated the therapy as “completely unacceptable” (i.e., 0). Nine participants agreed to take part in the qualitative interviews.

### Weekly outcome measures

#### Voice-related distress

At a group level, there was an overall significant reduction in distress between baseline and end of treatment (large effect; BC-SMD = − 1.02 (95% CI [− 1.81, − 0.24]). There was also a reduction from baseline to follow-up, although this was not significant and the effect size was small (BC-SMD = − 0.33 (95% CI [− 0.85, 0.20]).

**Fig. 2:**
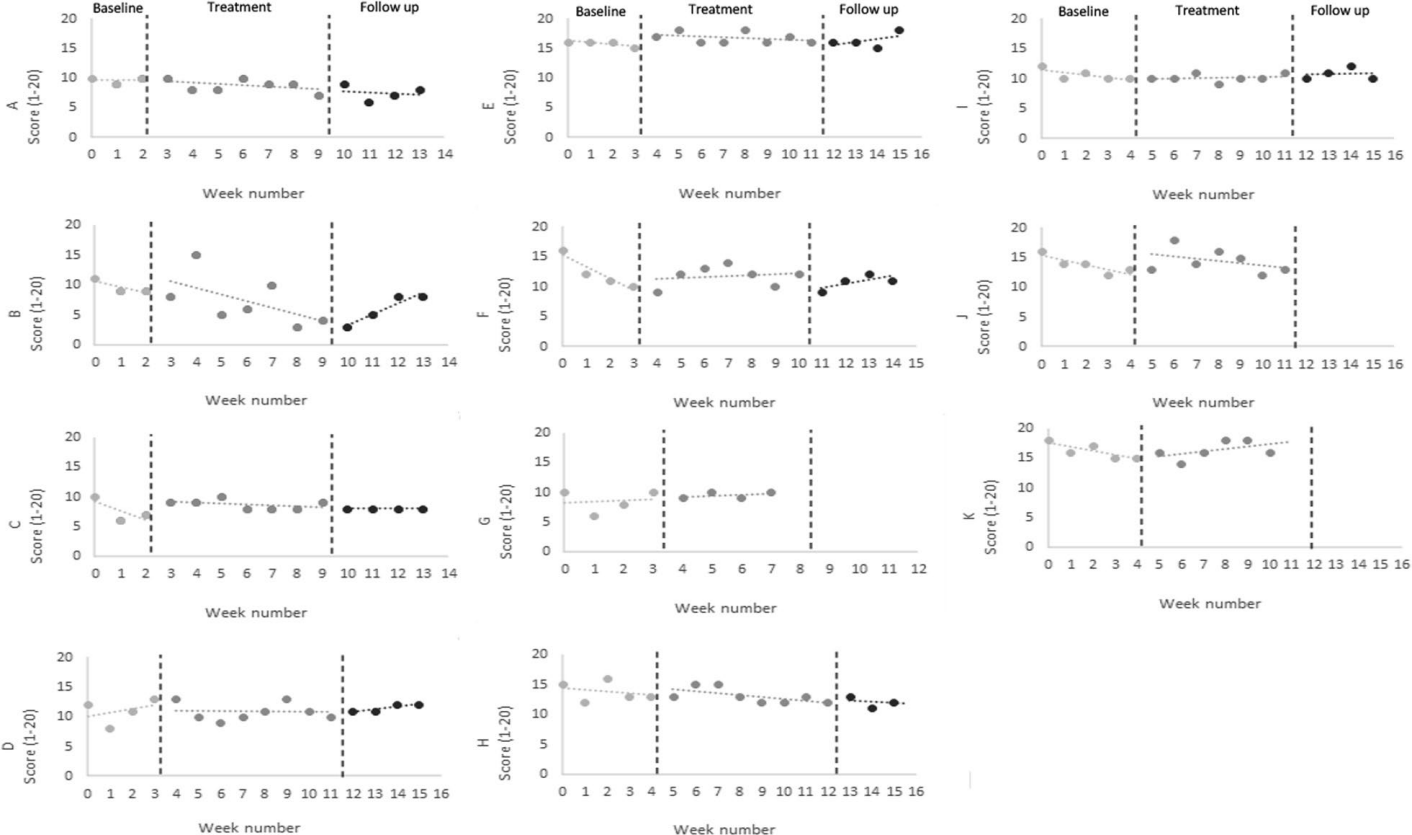
Participants’ individual scores on the PSYRATs Distress subscale over the baseline phase, therapy sessions and follow-up period

Based on individual-level data at the end of treatment, two participants showed both a reliable and a clinically significant decrease in distress; six achieved a reliable decrease and one a reliable increase (Table 1). Visual analysis of data (Fig. 2) indicated five patients had a stable baseline and a reliable reduction in mean distress from baseline to end of treatment. Two did not have a stable baseline but demonstrated a clinically significant reduction. From treatment to follow-up, five patients had a shift in distress scores; one showed a reliable increase and one a reliable decrease. At follow-up, the variation was small for all participants.

**Table 1:** Participants’ individual scores on the PSYRATS Distress subscale. Data expressed as means and standard deviation (SD) across the study’s phases

Participant	Baseline Mean (SD)	End of treatment Mean (SD)	Follow up Mean (SD)
A	11.00 (2.64)	10.29 (0.95)	10.50 (1.29)
B	11.33 (0.58)	8.00*** (3.51)	10.75 (0.96)
C	12.33 (1.15)	7.00***(2.83)	13.00 (0.00)
D	11.50 (1.73)	11.75(1.98)	14.00 + (0.00)
E	15.25 (0.50)	13.63* (1.77)	14.00 (0.00)
F	12.25 (1.50)	10.86 (1.22)	10.50* (1.29)
G	13.00 (2.95)	10.00* (2.71)	n/a
H	14.00 (0.70)	11.25* (2.66)	14.75 (1.50)
I	13.40 (0.89)	11.43* (1.40)	13.00 (0.82)
J	10.80 (2.28)	13.29 + (1.70)	n/a
K	16.20 (0.45)	14.00* (1.41)	n/a
Total	12.82 (1.76)	11.04* (2.20)	12.56 (1.74)

Maximum score = 20, Minimum score = 0

*n/a* data not available

*Reliable decrease

**Clinically significant decrease

***Reliable and clinically significant decrease

^+^reliable increase

### Voice frequency

At group level, changes between baseline and treatment (BC-SMD = 0.31 (95% CI [− 0.19, 0.81]) and baseline and follow-up (BC-SMD = 0.22 (95% CI [− 0.7729, 0.3552]) were not significant.

**Fig. 3:**
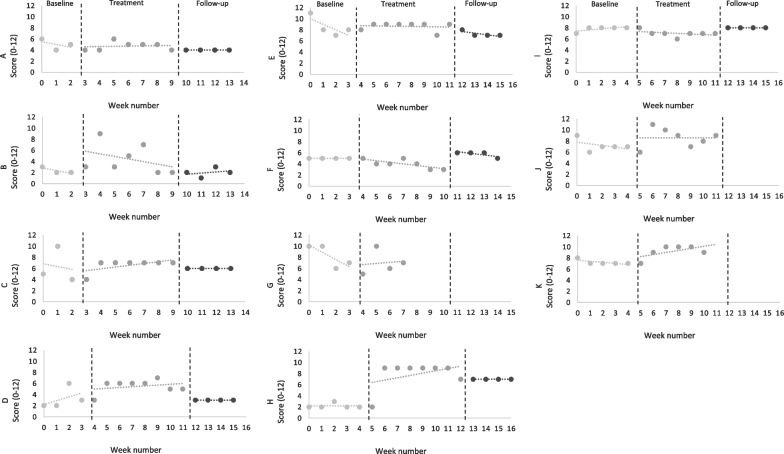
Participants’ individual scores on the PSYRATs Frequency subscale over the baseline phase, therapy sessions and follow-up period

Based on individual-level data at the end of treatment, no participants showed both a reliable and a clinically significant decrease in voice frequency; two achieved a reliable decrease and four a reliable increase (Table 2). Visual analysis (Fig. 3) indicated that seven participants had a stable baseline (63.6%), of whom two had an upward trend and two a downward trend across the treatment phase. The remaining three showed no trend on visual analysis. At follow-up, no participants showed a reliable or clinically significant change.

**Table 2:** Participants’ individual scores on the PSYRATs Frequency subscale. Data expressed as means and standard deviation (SD) across the study’s phases

Participant	Baseline Mean (SD)	Treatment Mean (SD)	Follow up Mean (SD)
A	5.00 (1.00)	4.71 (0.76)	4.00 (0.00)
B	2.33 (0.58)	4.43 + (2.70)	2.00 (0.82)
C	6.33 (3.21)	6.57 (1.13)	6.00 (0.00)
D	3.25 (1.89)	5.50 + (1.20)	3.00 (0.00)
E	8.50 1.73)	8.63 (0.74)	7.25 (0.50)
F	5.00 (0.00)	4.00* (0.82)	5.75 (0.50)
G	8.25 (2.06)	8.63 (2.16)	n/a
H	2.20 (0.45)	7.88 + (2.47)	7.00 (0.00)
I	7.80 (0.45)	7.00* (0.58)	8.00 (0.00)
J	7.20 (1.10)	8.57 + (1.72)	n/a
K	7.20 (0.45)	9.17 + (1.17)	n/a
Total	5.73 (2.32)	6.68 (1.81)	5.38 (2.15)

Maximum score = 20, Minimum score = 0

*n/a* data not available

*Reliable decrease

**Clinically significant decrease

***Reliable and clinically significant decrease

^+^reliable increase

### Brief 6-item eating disorder voice survey

**Fig. 4:**
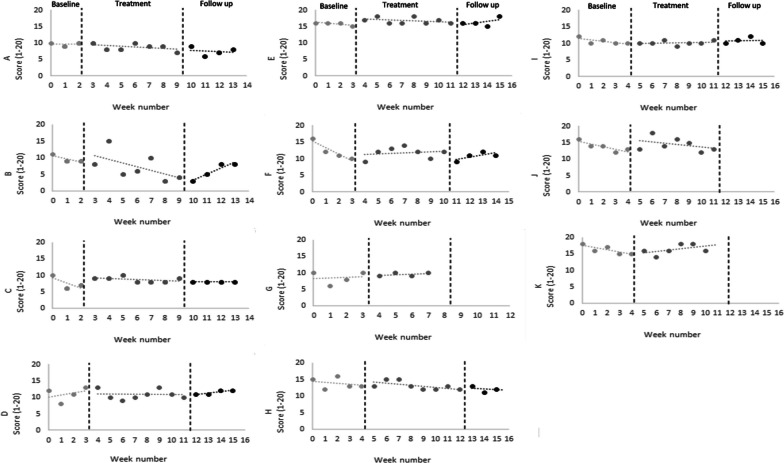
Participants’ individual scores on the Brief 6-item eating disorder voice survey over the baseline phase, therapy sessions and follow-up period

At group level, there was a small and not significant reduction in the brief 6-item measure between baseline and end of treatment (BC-SMD = − 0.06 (95% CI [− 0.31, 0.19) and between baseline and follow-up (BC-SMD = − 0.19 (95% CI [− 0.55, 0.17]). Individual data are described in Table 3. Visual analysis indicated that three participants had a stable baseline (27.3%), although none showed a specific trend during the treatment or follow-up phase (Fig. 4).

**Table 3:** Participants’ individual scores on the Brief 6-item eating disorder voice survey. Data expressed as means and standard deviation (SD) across the study’s phases

Participant	Baseline Mean (SD)	Treatment Mean (SD)	Follow up Mean (SD)
A	9.67 (0.58)	8.71 (1.11)	7.50 (1.29)
B	9.67 (1.15)	7.29 (4.15)	6.00 (2.45)
C	7.67 (2.08)	8.71 (0.76)	8.00 (0.00)
D	11.00 (2.16)	10.88 (1.46)	11.50 (0.58)
E	15.75 (0.50)	16.75 (0.89)	16.25 (1.26)
F	12.25 (2.69)	11.71 (1.70)	10.75 (1.26)
G	8.50 (1.91)	9.50 (0.58)	n/a
H	13.80 (1.64)	13.13 (1.25)	12.00 (0.82)
I	10.60 (0.89)	10.14 (0.69)	10.75 (0.96)
J	13.80 (1.48)	14.43 (2.07)	n/a
K	16.20 (1.30)	16.33 (1.51)	n/a
Total	11.72 (2.87)	11.60 (3.19)	10.34 (3.20)

### Pre, post and follow-up outcomes

Mean scores for the BAVQ-r, EDE-Q, DASS and SCSC questionnaires are described in Table 4. Self-compassion scores demonstrated a clinically significant change (i.e., an increase) at the end of the follow-up period. At post-treatment, mean scores for stress and anxiety dropped to the mild severity range from the moderate range; this was maintained at follow-up. Mean depression scores remained moderate.

**Table 4:** Voice’s characteristics, eating disorder psychopathology, symptoms of depression, anxiety and stress, and self-compassion and self-criticism at baseline, end of treatment and follow-up. Data expressed as means and standard deviations (SD)

	Baseline Mean (SD)	Treatment Mean (SD)	Follow up Mean (SD)
BAVQ-R Malevolence Scores 0–18	10.75 (4.45)	11.89 (2.62)	10.63 (3.93)
BAVQ-R Benevolence Scores 0–18	4.58 (4.60)	3.22 (3.56)	2.63 (2.50)
BAVQ-R Omnipotence Scores 0–18	12.25 (3.41)	10.78 (3.70)	9.25 (1.39)
BAVQ-R Resistance	15.92 (4.83)	17.89 (3.10)	18.88 (4.58)
Scores 0–27			
EDE-Q Scores 0–6	3.57 (1.27)	3.10 (1.50)	3.02 (1.36)
DASS Stress Scores 0–42	20.00 (7.63)	16.89 (6.57)	16.25 (6.80)
DASS Anxiety Scores 0–42	12.17 (8.07)	8.89 (6.72)	7.75 (6.54)
DASS Depression Scores 0–42	20.00 (15.89)	18.44 (6.72)	16.75 (6.54)
Self-Compassion Scores 15–105	23.95 (11.38)	30.33 (14.92)	32.13** (13.92)
Self-Criticism Scores 15–105	79.67 (17.24)	74.33 (13.74)	73.13 (16.92)

*Reliable decrease

**Clinically significant decrease

***Reliable and clinically significant decrease

^+^Reliable increase

### Qualitative data

Four superordinate themes emerged from participants’ accounts:

Theme 1: The first theme related to the impact of AVATAR therapy on clinical symptoms, with participants suggesting that the therapy had helped with standing up to the illness and making changes around eating as well as increasing their motivation to recover and self-compassion.

Theme 2: This theme emerged in relation to the core mechanisms of treatment which participants identified, such as the externalisation of the illness and the experience of validation, alongside the therapy being practical and goal oriented. Theme 3: This theme related to the characteristics of therapy delivery, such as it being personalised, time-limited and online.

Theme 4: This theme related to suggestions for future development of the intervention and included reflections on developing a staged-approach application of the therapy and the involvement of close others. A more detailed description of the superordinate themes and subthemes is provided in the Additional file 1. Table 5 describes the superordinate and subordinate themes which emerged from the data. A more detailed description of the themes is included in Additional file 1: Material 7.

**Table 5:** Superordinate and subordinate themes from a thematic analysis of participants’ experience of AVATAR therapy

Superordinate themes	Subordinate themes
Impact of AVATAR therapy on clinical symptoms	Standing up to the illness
	Increased motivation to recover
	Increased ability to express compassion towards the self and the illness
	Facilitated changes around food and eating disorder behaviours
Core mechanisms of treatment	Relating to the illness as external to the self
	Differentiation between personal values and the intentions of the illness
	Experiencing validation
	Therapy generalisation to real-world context
Characteristics of therapy delivery	Visualising the illness as a separate entity
	Goal oriented and personalised
	Time limited approach
	Online delivery
	Role within overall treatment
Suggestions for future developments of the intervention	Staged approach
	Involvement of close others

## Discussion

This study investigated the feasibility, acceptability and safety of an adaptation of AVATAR therapy for patients with anorexia nervosa. A preliminary analysis of the impact of the intervention on the eating disorder voice, eating disorder psychopathology, anxiety, stress and depression symptoms, self-compassion and self-criticism was also conducted. AVATAR therapy for anorexia nervosa appeared feasible, as demonstrated by the timely recruitment of the target sample and a retention rate of over 90%. No adverse experiences associated with the intervention were reported or observed and over 80% of patients rated the intervention as seven or above in satisfaction. Among the variables assessed, voice-related distress decreased, and self-compassion increased significantly after the therapy. No other significant changes in outcomes were observed.

AVATAR therapy was an approach first developed as an intervention for distressing voices in psychosis (typically experienced by voice-hearers as originating from a source external to the self). The therapy engagement and completion rates in this feasibility work compare favourably to the larger trials of AVATAR therapy for psychosis where rates of just over 70% have been reported [28]. In keeping with the ongoing multi-site trial of AVATAR Therapy for psychosis [27], the preliminary findings from this study suggest that voice-related distress would be a key outcome to explore in future randomised trials of AVATAR therapy for eating disorders. Voice-frequency appears a less helpful metric of change given that (in contrast to voices in psychosis) an experience of voice frequency might paradoxically increase the more the person separates from the eating disorder. The clinically significant increase in self-compassion at the end of the follow-up is encouraging given the role of harsh self-criticism in eating disorders [42, 43]. This finding is also relevant considering that self-compassion has been found to protect against eating disorder related concerns (i.e., eating, body image; Braun, Park, Gorin, 2016) and to be associated with lower eating disorder psychopathology (Turk, Waller, 2020). A possible mechanism for the positive impact of self-compassion on eating disorder symptoms is the regulatory effect that it exerts on negative emotions (Neff, 2003). This is because individuals’ ability to regulate negative affect more efficiently might reduce the need to use dangerous eating disorder behaviours (Gilbert, 2009). Future work is needed to replicate the finding of increased self-compassion following AVATAR therapy in anorexia nervosa and to investigate potential mechanisms of effect. The use of self-report measures more specifically measuring this construct, as opposed to measuring self-criticism would be particularly helpful.

The qualitative analyses highlighted that participants reported an increased ability and confidence to assert themselves over the eating disorder voice. This translated into having the active choice to engage with the voice or not and the power to challenge it and “act opposite”. Patients identified two main mechanisms for the perceived change. The first mechanism was the externalisation of the illness (i.e., considering the illness as a distinct entity compared to the self). This process was perceived as highly validating and helpful in developing a discrepancy between the self and the eating disorder. The work on externalisation as well as the identification of personal values and goals helped participants to realise how the eating disorder was getting in the way of living according to their values. The second mechanism of change was in the ability to express positive emotions towards the self, which for some, was the most challenging part of the therapy [42, 43]. Interestingly, some patients took the step of sharing dialogues with loved ones (as has been the case in psychosis work) as a means of sharing their inner experience and fostering empathic understanding across the family system.

When considering future developments of AVATAR therapy, some patients highlighted the need for more sessions and a possibly adaptations based on the characteristics of the different stages of the illness. They indicated, for example, that delivering AVATAR therapy when the existence of an eating disorder voice is not acknowledged by the individual would be more harmful than beneficial. Also, they felt the importance to deliver it at a stage when people are ready to take responsibility for their own recovery, rather than in a more ambivalent phase. Finally, participants highlighted the relevance of involving significant others in the therapy to help with behaviour change outside the therapy sessions. Offering more sessions, at “the right stage” and involving others might therefore contribute to sustain the benefits of AVATAR therapy in the longer term. This is important because when changes in voice-related distress occurred in this study, they seemed to fade away at follow-up. Similarly, although the qualitative feedback described relevant changes of the eating disorder psychopathology, these were not captured by the validated questionnaire that we selected (the EDE-Q). Larger samples are needed to test changes on the quantitative measures. At the same time, the use of assessment measures that capture facets of the disorder more directly linked with the eating disorder voice (e.g., patients’ beliefs on their eating disorder thoughts) might be needed. Finally, changes to the therapy protocol that involve exercises around bringing dialogue with the voice “to life”, when needed (e.g., at mealtimes or when observing one’s reflection in the mirror), could strengthen changes in the core psychopathology.

### Strengths and limitations

This study represents an important step forward in testing AVATAR therapy in eating disorders. Strengths include the use of a SCED and the embedding of qualitative interviews to provide convergent evidence of acceptability. However, it should be noted the two people who did not engage with the qualitative interviews included the one therapy drop-out (despite attempts to include this person). Therefore, the qualitative analysis may have missed important aspects relating to challenges in the approach and barriers to engagement. Future work should include purposive sampling of people who have disengaged from therapy to address this limitation. In addition, it is important to note that the study was not powered to answer questions about clinical efficacy and presentation of quantitative outcomes should be interpreted as preliminary and in need of replication in fully powered studies. Some measures, and especially the 6-item voice measure, did not show stability over time. This might be due to the poor psychometric properties of the scale (which is not validated and that was developed on purpose, for this study) and/or to the large variability of the experience of the voice among participants. Future studies might want to include assessment measures which are validated and that are specifically designed to capture the nuanced, at times ambivalent and changeable relationship that patients with anorexia nervosa have with the eating disorder voice. Since the time the trial was conducted, a novel questionnaire became available for this purpose, the Experience of an Anorexic VoicE Questionnaire (EAVE-Q; Hampshire et al., 2020). More specific assessment measures, together with the implementation of a randomised controlled trial are among the most useful steps to understand the potential of AVATAR therapy in anorexia nervosa. Ongoing consultation with people with lived experience is also warranted to define therapeutic processes and outcomes. Some patients in this study indicated that the inclusion of food exposure tasks in the therapy might be helpful to embed and sustain change over time. This is an interesting proposal which deserves future attention. Finally, the recruitment of patients with anorexia nervosa in clinical studies is notoriously difficult (e.g., Phillips et al., 2023). Our experience in this field is that patients are very reluctant to engage and remain in therapy. Also, recruitment for this study and AVATAR therapy took place online, at distance, which adds another layer of complexity to the implementation of the study. For this reason and considering that this study was aimed at testing the feasibility (rather than the efficacy/effectiveness) of AVATAR therapy, we chose a conservative threshold to establish the feasibility of recruitment. To our surprise, the target sample was recruited within a much shorter time window than what we had planned. It is an interesting proposal that this might reflect patients’ willingness to try out new therapy approaches.

## Conclusion

This study presents a crucial step in developing AVATAR therapy as a novel intervention for eating disorders. It provides encouraging evidence of feasibility, acceptability and safety with qualitative feedback suggesting potential for important clinical impact. The next steps will be to work collaboratively with people with lived experience of the illness, including recovered individuals, patients, carers and professionals to further develop this approach.

### Supplementary Information

**Additional file 1**. Justification for the choice of the study design, interview schedule, brief 6-item eating disorder voice survey, therapy aims and contents and themes from qualitative analysis.

## Data Availability

The datasets used and/or analysed during the current study are available from the corresponding author on reasonable request.
