# Supplementary material for: A case series to test the acceptability, feasibility and preliminary efficacy of AVATAR therapy in anorexia nervosa
Source: J Eat Disord. 2023 Oct 13;11:181. doi: 10.1186/s40337-023-00900-1 (PMC10571357; doi:10.1186/s40337-023-00900-1)
Supplement: Supplementary file 1 — Additional file 1. Justification for the choice of the study design, interview schedule, brief 6-item eating disorder voice survey, therapy aims and contents and themes from qualitative analysis. [file 40337_2023_900_MOESM1_ESM.docx]

**Supplementary materials**

*Supplementary Material 1*

Non-concurrent multiple baselines single case design

Non-concurrent multiple baselines single case designs (SCED) encompass a collection of methods for testing hypotheses with a small number of participants, such as feasibility studies. The inclusion of multiple baseline designs helps control for threats to validity such as maturation and statistical regression (i.e., regression to the mean) when treatment reversal is not possible, from a clinical or ethical perspective. Confidence in the effect of the intervention arises from change occurring in a dependent variable once the intervention is introduced following a stable baseline period. The effect should be replicated in a minimum of three cases at three distinct time points to have confidence that the effect is not the result of an extraneous variable (i.e., maturation or a coincidental event) (Ferron et al., 2014; Horner et al., 2016; Kratochwill et al., 2013).

*Supplementary Material 2*

Interview schedule

- How did you find the experience of Avatar Therapy overall?
- How did it work for you to receive the therapy remotely?
- What did motivate you to take part?
- How did the dialogue with the AVATAR influence your understanding of the eating disorder voice?
- How did the dialogue with the AVATAR influence your relationship with the eating disorder voice?
- How did the therapy impact on your eating disorder?
- What do you think about the impact of the therapy at a different stage of recovery?
- How did the therapy impact on how you see yourself in the recovery process?
- How does AVATAR therapy compare to other treatments you have received?
- How would you change AVATAR therapy to support yourself in recovery?
- How do you think significant others could be involved in AVATAR therapy?

*Supplementary Material 3*

Brief 6-item eating disorder voice survey

Please answer the following questions with reference to the previous 7 days

*How often did the eating disorder voice make negative comments about yourself (i.e. contents not specifically related to your eating disorder)?*

0 Not at all

1 Sometimes

2 Often

3 All of the time

*How often did the eating disorder voice make negative comments specifically related to the need to avoid or restrict food intake?*

0 Not at all

1 Sometimes

2 Often

3 All of the time

*How often did the eating disorder voice make negative comments specifically related to the need to compensate for what you had eaten (e.g. through over-exercising, purging, using laxatives or diuretics)?*

0 Not at all

1 Sometimes

2 Often

3 All of the time

*How often did the eating disorder voice make negative comments specifically related to your body, weight, physical appearance?*

0 Not at all

1 Sometimes

2 Often

3 All of the time

*To what degree did the eating disorder voice make negative comments related to your social relationships (e.g. commenting that you are alone, or unable to connect to others, or that others do not care about you)?*

0 Not at all

1 Sometimes

2 Often

3 All of the time

*Please circle the number which best describes how you feel in relation to your voice.*

1 I am much more powerful than my voice

2 I am more powerful than my voice

3 We have about the same amount of power

4 My voice is more powerful than me

5 My voice is much more powerful than me

1 I am much more powerful than my voice

2 I am more powerful than my voice

3 We have about the same amount of power

4 My voice is more powerful than me

5 My voice is much more powerful than me

*Supplementary Material 4*

Supplementary Table 1. AVATAR Therapy for anorexia nervosa: sessions’ aims and contents

| **SESSION** | **AIM** | **CONTENT** |
| --- | --- | --- |
| ***Session 0 (assessment)*** | Assessment of ED voice, including verbatim content, and avatar creation. | To develop an understanding of the experience of the ED voice, including characteristics of the voice, power, triggers, impact on functioning, emotional experience. To identify verbatim content most often spoken by the voice. To create the computerised avatar by manipulating voice and face characteristics. |
| ***Session 1*** | To engage in active dialogue with the avatar practicing calm assertiveness. The dialogue is facilitated to end on a “win”. | To explore and practice calm assertiveness in responding to the voice by supporting participant to identify an assertive role model and generate some assertive responses to the voice. The participant engages in first active dialogue with the avatar, ending the dialogue with a minor win. |
| ***Session 2*** | To build assertiveness (i.e. power and control) and drop avoidance and safety behaviour. Avatar pushes back before making a graded concession. | The participant builds assertiveness by reflecting on role of the voice in their life (e.g. a bully; critical). They work on assertiveness (e.g. tone of voice; eye-gaze; posture) and drop any safety behaviours. The avatar makes a concession by noticing a change in the participant’s responding. |
| ***Session 3*** | To practice assertiveness from standing in a confident posture. Consolidating sense of power and control. Avatar voicing clear concession. | The participant builds on prior achievements and practices talking to the avatar by standing in a confident posture. The avatar voices a clear concession by noticing the change in the participant and how they are seeing them in a different light. |
| ***Session 4*** | To explore personal qualities and build a narrative around positive change. Full avatar concession. | The focus shifts towards improvements in self-concept, self-esteem and identity. This dialogue is identified as an opportunity for the participant to communicate who they really are as a person and how they plan to move forward. A rationale for the voices leaving the person alone (or having a diminishing role in their life) starts to emerge. |
| ***Session 5*** | To continue work around self-concept and self-esteem. Avatar exerting reduced control and power. | Emphasis is on building and consolidating positive changes in self-concept and self-esteem. The avatar acknowledges the participant’s personal qualities and delivering a full concession (e.g. I have nothing left to say; if you continue to do X, my power is fading). |
| ***Session 6*** | To review and consolidate positive changes. To generalise learning to daily life. | The main themes which emerged and positive changes are reviewed and consolidated. The participant communicates a plan for moving forward and the avatar voices the fundamentally changed nature of the relationship and says goodbye. |

*Supplementary Material 5*

Supplementary Table 2. Reliable Change Index and Clinically Significant Change: cut-off for the outcomes considered in the study

| **Measure** | **RC index** | **CS cut-off** |
| --- | --- | --- |
| **PSYRATs Distress^[[1]](#footnote-1)^** | +/- 1.61 | Below 9.87 |
| **PSYRATs Frequency^[[2]](#footnote-2)^** | +/- 0.99 | Below 1.17 |
| **EDE-Q^[[3]](#footnote-3)^** | +/- 1.43 | Below 2.59 |
| **BAVQ-R Malevolence^[[4]](#footnote-4)^** | +/- 5.90 | Below 1.84 |
| **BAVQ-R Benevolence^5^** | +/- 3.68 | n/a |
| **BAVQ-R Omnipotence^5^** | +/- 3.24 | n/a |
| **BAVQ-R Resistance^5^** | +/- 3.12 | Above 25.58 |
| **DASS Stress^[[5]](#footnote-5)^** | +/- 8.45 | Below 14 |
| **DASS Anxiety^6^** | +/- 7.77 | Below 7 |
| **DASS Depression^6^** | +/- 6.81 | Below 9 |
| **SCSC Self Compassion^[[6]](#footnote-6)^** | +/- 13.97 | Above 28.46 |
| **SCSC Self Criticism^7^** | +/- 18.59 | Below 66.94 |

*Supplementary Material 6*

Additional information around participants’ safety during the study

One participant became distressed during session 4 when behavioural change was suggested by the therapist but continued to engage with the therapy. Participant H reported a preference to not engage with the dialogue during session 3 but did reengage with the dialogue in the following session and completed all 6 active dialogues. Participant J completed 5 active dialogues sessions and reported positive engagement and impact. However, after initially rescheduling a final planned ending session they became non-contactable. Attempts were made to contact the person by phone, text and email without success. Contact with next of kin (established routinely at the start of therapy) confirmed that they had contact with the participant and that there had been no serious adverse events. Finally, participant K, opted for five sessions of active dialogue rather than six and cited the demands of the therapy as the reason, the participant was also engaged with a day programme at the time.

*Supplementary Material 7*

Description of themes emerged from participants’ feedback of the therapy

- Theme 1: Impact of AVATAR therapy on clinical symptoms

A superordinate theme which emerged from the data related to the range of impacts of AVATAR therapy which participants identified. By creating a representation of their ED voice and engaging in a dialogue with it, AVATAR therapy supported participants to gain more power and control over the voice. All participants reported that the therapy improved their ability to stand up to the voice and appreciate that they had a choice in whether to engage with the voice and follow its commands. Participants reported learning ways to stand up to the voice [Q2] and feeling more empowered to “*be more dismissive* *[towards the voice]*” and “*to just ignore it*”. Additionally, most participants reflected on how the therapy boosted their motivation to recover and increased their confidence that their lives could be better [Q3]. “*The therapy made me realize how far I've come*” and it “*highlighted the areas where I particularly struggle with the ED*” and on which participants needed to focus to move forward in their recovery. AVATAR therapy also sought to support participants to foster self-compassion, with participants reflecting on their personal qualities and values in life. This helped to increase their compassion towards themselves [Q4] and be less self-critical: *“I shouldn't always beat myself up if I don't do well the first time, I can always come back to it again”.* Participants found *“this helpful, but that was definitely the most difficult part”.* A few participants also reported that the therapy allowed them to express anger towards the ED: *“I can be angry at it, I can just I can swear at it. I don't have to be kind to it”.* Participants described that by being more able to shut down the voice, they were able to make changes around food and eating. One participant engaged in food exposure in front of the hostile avatar [Q6] and managed to eat a gingerbread man. Many participants suggested that the therapy facilitated ‘*decision-making in the moment*’ where previously the ED would make an automatic choice. They now had ‘enough’ mental space to make an active choice [Q7], feeling more prepared to decide on food, even in social situations, rather than having a long debate with the voice.

- Theme 2: Core mechanisms of treatment

A second subordinate theme which emerged from the data was related to the aspects of treatment which supported and contributed to the changes described in the above section. Participants found the process of “*externalisation*”, that is separating the illness from the person by creating a visual representation of it, “*helpful, insightful and empowering*” and reported that it was something they struggled to do prior to engaging in the therapy. Relating to the voice as external to the self, made it easier to challenge the ED and to perceive it as something which could be changed: “*It doesn't feel so much like it's all in my head and like something that I can't escape from. I can give it a bit of distance”.* Externalising the ED seemed to be a validating experience for participants: “*the therapy validated my experience and made me feel that what I'd been going through is real*”. This process also opened opportunities for validation in the broader social network (e.g., family, friends). Some participants showed the recordings of the sessions to their significant ones to allow them *“to gain a better insight into what we experience”.* An important part of AVATAR therapy was to support individuals to reflect on their values, goals in life and identity and how their relationship with the ED voice fitted with them. Participants were encouraged to reflect on the nature and motives of the ED voice to gradually build more awareness of them. This was a theme which strongly emerged from the data, with most participants reporting that the therapy created an internal dissonance between what they identified as their values and what they came to understand as the true intentions of the ED voice [Q9]. Participants reported a realisation of how the ED tried to get in the way of their values and goals, across different areas, from social to academic life, and how “*my eating disorder isn't really my friend. It's something that's holding me back. It's something that's dominating my life”*. Another core aspect of treatment which was identified by participants related to the therapy being “*practical*” to support them *“to come up with some skills in terms of day-to-day management of the eating disorder”.* Participants used the skills learnt in therapy when facing situations around food [Q12] and they also noticed that doing the sessions remotely from home in the same environment where they faced the challenges was helpful and made it easier to generalise the skills learnt.

- Theme 3: Characteristics of therapy delivery

Participants provided feedback on different aspects of how the therapy was delivered. A crucial part of AVATAR therapy is the creation of the avatar; some participants found this process *“easier and more helpful than expected”,* whereas others found this process difficult, strange or odd, especially in the initial sessions, as they did not have an idea of *“what the avatar sounded like and looked like”.* A positive aspect of the therapy identified by participants was how AVATAR therapy specifically targeted the ED voice and how the goals set for the sessions and the dialogues were very individualised to their specific experience, using specific food-related situations and verbatim of the voice recalled by participants [Q15; Q16]. Participants reflected on the benefits and challenges of engaging in a time limited therapy. Some participants reported that 6 sessions were enough and that engaging in a time-limited approach increased their motivation [Q17]; however, most participants reported that 6 sessions *“was far too short”*, and that a longer therapy, *“maybe 12 sessions”,* would have been better [Q18]. An aspect of the therapy delivery on which all participants agreed was how they found participating in the therapy remotely, highly acceptable [Q19]; this allowed greater flexibility in scheduling the session [Q20] and made it easier to generalise the skills learnt in their home environment. Participants were positively surprised how there were “no *technical issues”* accessing the therapy; the only issue raised by a couple of participants was related to the privacy and safeguarding aspect of remote therapy, which would be problematic for risky and severely underweight participants. Lastly, participants reflected on how AVATAR therapy compared to other therapies they had received, and how they viewed it as a potential supplementary or complementary approach. Some participants viewed AVATAR therapy as *“something completely different”* which could be combined with other therapies such as CBT or MANTRA^[[7]](#footnote-7)^. There were different views on whether AVATAR therapy should be conducted alongside other therapies or as a standalone treatment, with some participants suggesting that it would be better *“to concentrate just on one form of treatment at the time”* [Q23]*,* and others saying that it worked well for them to have it *“alongside the other treatment I was having”* [Q24]*.*

- Theme 4: Suggestions for future developments of the intervention

A last superordinate theme which emerged from participants’ accounts related to their views on future developments of AVATAR therapy. Participants reflected on the timings for implementing the therapy, sharing their opinions on the potential advantages and disadvantages of applying the therapy at different stages according to their experience and recovery journey. A large proportion of participants suggested that the therapy could be applied in earlier stages of the illness [Q25], whereas others suggested that “i*t could be so valuable at any stage”* [Q26]. Concerns were raised regarding applying the therapy with individuals who did not experience an ED voice, suggesting that *“it would have potentially made things worse for me as I experienced thoughts at the beginning”* and that might be invalidating for individuals: “*I may have thought that there was something wrong with me because I didn’t experience it as a voice”.* It was widely suggested that to successfully implement this therapy, individuals needed to be at a stage where they take responsibility for their recovery and *“they have to be willing to engage, if they're not motivated to recover, then there's no point*”. Participants also considered the value of involving significant others in the therapy, to support them to generalise the skills learnt to challenge the voice [Q27], especially “*if you're having a particularly difficult moment or at mealtimes*” and need some encouragement.

1. Internal consistency of the measure obtained from Noordenbos et al. (2014) and standard deviation of comparative sample obtained from an AN sample (Cardi et al., personal communication). [↑](#footnote-ref-1)
2. Internal consistency of the measure obtained from Noordenbos et al. (2014) and the standard deviation of comparative sample obtained from AVATAR for psychosis trial (Craig et al., 2018) as PSYRATs auditory subscales data within an eating disorder sample was not available. [↑](#footnote-ref-2)
3. Internal consistency of the measure obtained from the mean of several eating disorder studies (Ekeroth & Birgegård, 2014) and the standard deivation of a comparative sample obtained from eating disorder norm data in the UK (Carey et al., 2019). [↑](#footnote-ref-3)
4. Internal consistency of the measure obtained from the use of the subscales in eating disorder samples (Noordenbos et al., 2014; Noordenbos & Geest et al., 2017). The standard deviation of comparative sample obtained from psychosis sample (Chadwick et al., 2000) as data from an eating disorder sample unavailable. [↑](#footnote-ref-4)
5. Internal consistency & standard deviation data obtained from Sinclair et al. (2011). [↑](#footnote-ref-5)
6. Internal consistency & standard deviation data obtained from Falconer et al. (2015). [↑](#footnote-ref-6)
7. MANTRA Maudsley Anorexia Nervosa Treatment for Adults [↑](#footnote-ref-7)
